# Barriers and facilitators for healthcare professionals to the implementation of Multidisciplinary Timely Undertaken Advance Care Planning conversations at the outpatient clinic (the MUTUAL intervention): a sequential exploratory mixed-methods study

**DOI:** 10.1186/s12904-023-01139-y

**Published:** 2023-03-15

**Authors:** Eline V. T. J. van Lummel, Yoeki Meijer, Dave H. T. Tjan, Johannes J. M. van Delden

**Affiliations:** 1 Department of Intensive Care, Gelderse Vallei hospital, Ede, Netherlands; 2 Julius Center for Health Sciences and Primary Care, University Medical Center Utrecht, Utrecht, Netherlands

**Keywords:** Advance care planning, Outpatient clinic, Palliative care, Implementation, Healthcare professional, Barrier, Facilitator, Mixed-methods research

## Abstract

**Background:**

Advance Care Planning (ACP) enables patients to define and discuss their goals and preferences for future medical treatment and care. However, the structural implementation of ACP interventions remains challenging. The Multidisciplinary Timely Undertaken Advance Care Planning (MUTUAL) intervention has recently been developed which takes into account existing barriers and facilitators. We aimed to evaluate the MUTUAL intervention and identify the barriers and facilitators healthcare professionals experience in the implementation of the MUTUAL intervention and also to identify suggestions for improvement.

**Methods:**

We performed a sequential exploratory mixed-methods study at five outpatient clinics of one, 300-bed, non-academic hospital. Firstly, semi-structured interviews were performed with a purposive sample of healthcare professionals. The content of these interviews was used to specify the Measurement Instrument for Determinants of Innovations (MIDI). The MIDI was sent to all healthcare professionals. The interviews and questionnaires were used to clarify the results.

**Results:**

Eleven healthcare professionals participated in the interviews and 37 responded to the questionnaire. Eight barriers and 20 facilitators were identified. Healthcare professionals agreed that the elements of the MUTUAL intervention are clear, correct, complete, and simple - and the intervention is relevant for patients and their proxies. The main barriers are found within the user and the organisational domain. Barriers related to the organisation include: inadequate replacement of staff, insufficient staff, and insufficient time to introduce and invite patients. Several suggestions for improvement were made.

**Conclusion:**

Our results show that healthcare professionals positively evaluate the MUTUAL intervention and are very receptive to implementing the MUTUAL intervention. Taking into account the suggestions for improvement may enhance further implementation.

**Supplementary Information:**

The online version contains supplementary material available at 10.1186/s12904-023-01139-y.

## Introduction

Advance Care Planning (ACP) is defined as: “*enabling individuals to define goals and preferences for future medical treatment and care, to discuss these goals and preferences with family and healthcare providers, and to record and review these preferences if appropriate*” [1]. ACP can have varying underlying goals, including supporting patient autonomy, improving the quality of care, strengthening relationships, preparing for the end of life and reducing overtreatment [2].

Various attempts to implement ACP interventions in various settings have been made, including interventions at paediatric care units [3], general practices [4–7], nursing home cares [8], and hospital settings [9]. However, the structural implementation of ACP interventions remains challenging. De Vleminck et al. describe four features for successful ACP interventions including 1) using a trained or experienced facilitator; 2) introducing a selection process to identify eligible patients; 3) having structured and patient-centered ACP discussions, and; 4) having the opportunity to complete ACP documentation [4]. The key factors for successful implementation of ACP interventions are trained staff, a structured approach, and organisational support [10].

The Multidisciplinary Timely Undertaken Advance Care Planning (MUTUAL) intervention was developed in 2018, which takes into account the existing barriers to, and facilitators for, successful ACP interventions [11]. The MUTUAL intervention consists of four steps: 1) timely patient selection; 2) the preparation of the patient and healthcare professionals; 3) a scripted ACP conversation in a multidisciplinary setting, and; 4) documentation. In this context, multidisciplinary refers to the involvement of nurses facilitating the first part of the ACP conversation and medical specialists joining in the second part of the ACP conversation. In the Netherlands, medical specialists are board-certified physicians. In this article, we refer to the medical specialists as treating physicians. The MUTUAL intervention was shown to be feasible and considered valuable by patients and healthcare professionals [11]. Subsequently, in 2019, the ACP intervention was implemented at the five different outpatient clinics comprising pulmonology, geriatrics, cardiology, oncology, and nephrology of one, 300-bed, non-academic hospital in the Netherlands.

According to Vanderhaeghen et al., several barriers for hospital physicians to engage in ACP exist. These include: lack of communication skills; a lack of knowledge concerning ACP; a lack of time; cultural differences; and the fear of medico-legal repercussions [12]. The lack of structural implementation of ACP is also perceived as a barrier [12]. Vanderhaeghen et al. stress the importance of knowing about the barriers and facilitators for healthcare professionals in a hospital setting if it is to be successfully implemented [12]. Hence, a greater understanding of these barriers and facilitators to the implementation of the MUTUAL intervention can facilitate structural implementation.

The aim of this study was to evaluate the intervention and identify the healthcare professionals’ perception of the barriers to, and facilitators for the implementation of the MUTUAL intervention recently developed at the outpatient clinic. Secondly, we want to identify suggestions for improving the implementation of the MUTUAL intervention.

## Methods

**Fig. 1:**
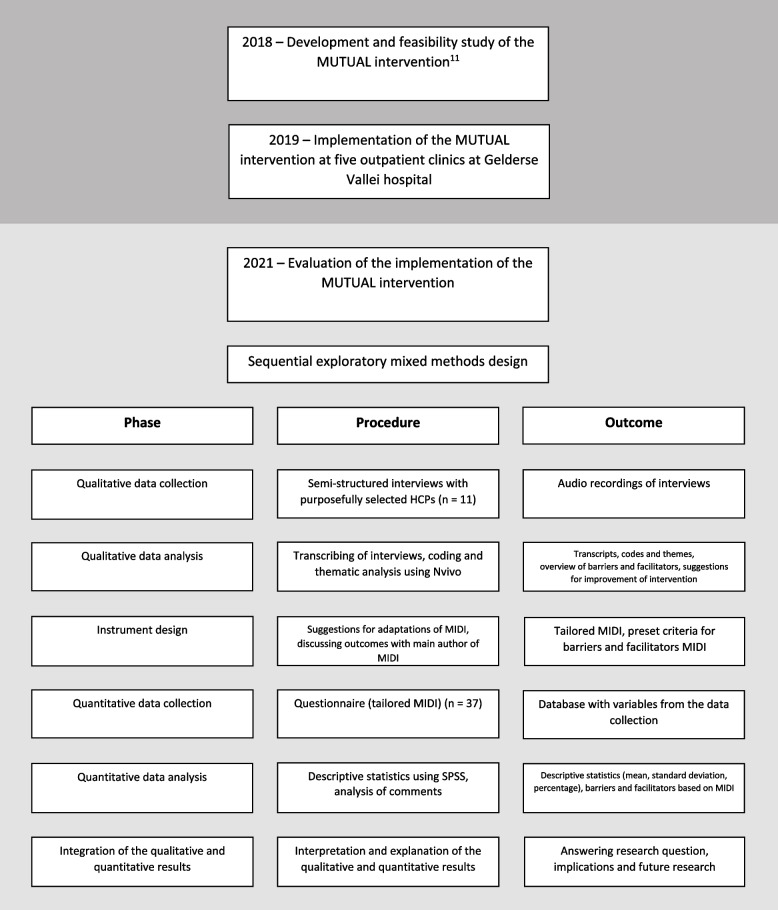
Flowchart displaying the timeline of the previous study and the implementation (development & feasibility study and subsequent implementation, dark grey) and the design of the current study (light grey). MIDI: Measurement Instrument for Determinants of Innovations; MUTUAL: Multidisciplinary Timely Undertaken Advance Care Planning; HCPs: Healthcare professionals

The Consolidated Criteria for Reporting Qualitative Research (COREQ) were used for optimising reporting [13]. We performed a sequential exploratory mixed-methods study to evaluate the recently developed MUTUAL intervention and to identify the barriers to, and facilitators for, the implementation of the MUTUAL intervention at five outpatient clinics of one, 300-bed, non-academic hospital in the Netherlands. A flowchart displaying the timeline of the previous study and the implementation (dark grey), and the design of the current study (light grey) can be found in Fig. 1.

### Description of the MUTUAL intervention

The development and feasibility study of the MUTUAL intervention has been performed previously (2018) at Gelderse Vallei hospital [11]. The ACP intervention developed consists of four steps. An elaborate description of the development and feasibility study of the MUTUAL intervention can be found in van Lummel et al. (2022) [11]. This intervention was implemented at five outpatient clinics covering different specialties comprising pulmonology, geriatrics, cardiology, oncology, and nephrology.

### Study design and participants

We performed a sequential exploratory mixed-methods study consisting of semi-structured interviews and a questionnaire. The Measurement Instrument for Determinants of Innovations (MIDI) [14] (a questionnaire) was used to evaluate the implementation of the MUTUAL intervention. There were two reasons for performing semi-structured interviews. Firstly, to identify the barriers to, and facilitators for, implementation in order to draw up a specification for the MIDI and thus maximise its use. Secondly, in order to clarify the results, we wanted to integrate the content of the interviews, the comments on the questions contained within the MIDI, and the answers to the open-ended questions. The research team consisted of two qualitative researchers with a medical background (EvL, JvD), a senior medical student (YM) and an intensive care physician (DT). Moreover, JvD is a professor in medical ethics.

Firstly, semi-structured in-depth interviews were conducted at Gelderse Vallei hospital by YM (female, medical student) between September 10, 2021, and September 24, 2021. The interviews were conducted in Dutch and the interviewer did not have any relationship with the interviewees before the study. No repeat interviews were carried out. The interview guideline was piloted with EvL and DT. The quality of the interviews was checked throughout the process by an evaluation and feedback session after each interview and by listening to the audio recordings. Participants were informed of the profession of the interviewer (YM) and the aim of the research. Fourteen healthcare professionals (nurses and physicians) from the five outpatient clinics that implemented the MUTUAL intervention were purposively invited to participate by email. At the geriatrics department the role of the treating physician can be replaced by a physician assistant and a physician assistant was therefore also invited. We aimed to include healthcare professionals 1) experienced with the MUTUAL intervention, varying from somewhat experienced to very experienced, and; 2) from all five outpatient clinics to ensure that no relevant barriers and facilitators were overlooked when specifying the MIDI and also to be able to consider potential differences between the different outpatient clinics.

Subsequently, the specified MIDI was sent to all healthcare professionals who could potentially participate in the MUTUAL intervention. Hence, invitations were sent to 1) nurses who had received training to be able to facilitate the ACP conversations, and; 2) physicians from the five outpatient clinics that had implemented the MUTUAL intervention. We invited all healthcare professionals working at an outpatient clinic that had implemented the MUTUAL intervention in the organisation of their specialty. This was done in an attempt to prevent inclusion bias and to gain the full scope of barriers to, and facilitators for, the implementation of the intervention. Invitations were sent using email. All eligible healthcare professionals were requested to fill in the online questionnaire using the hospital’s digital questionnaire system [15]. Those not responding to the questionnaire received two reminders. Data collection took place between November 2021 and January 2022, that is approximately two and a half years after the start of the implementation of the MUTUAL intervention. Due to the way in which the study was set up, healthcare professionals participating in the interviews were also invited to participate in the questionnaire.

### Data collection

#### Interviews

To ensure all relevant factors influencing the implementation of the intervention were discussed during the interviews, interview guidelines were created based on 1) the four elements of the MUTUAL intervention, and; 2) the determinants of the MIDI. Separate interview guidelines were created for nurses and physicians due to their different roles within the intervention. The interview guidelines are available in Supplementary file 1 (interview guideline for nurses) and Supplementary file 2 (interview guideline for physicians). The four different components of the intervention were discussed during the semi-structured interviews. The interviews were audio recorded, transcribed verbatim, and anonymised. Summarising field notes were made after each interview. The transcripts were not returned to the participants for comments or corrections.

#### MIDI

According to the MIDI, the factors related to the process of implementation are related to the innovation (the MUTUAL intervention), the user (patients and healthcare professionals), the organisation (hospital) and the socio-political context [14]. The original questionnaire consists of 29 determinants with response scales ranging from 1 (‘totally disagree’) to 5 (‘totally agree’). Adjustments to the MIDI were based on the interviews and made after consultation with the main author of the MIDI, Dr. M. Fleuren. In addition to the determinants of the MIDI, questions were added addressing the background of the participants, including their profession, medical specialty, years of experience in their current profession, and experience with the MUTUAL intervention.

### Analysis

#### Interviews

A thematic analysis [16, 17] was performed to identify the barriers and facilitators per domain of the MIDI (intervention, user, organisation, and socio-political context). The thematic analysis consisted of three phases: 1) creating a code tree; 2) coding the transcripts, and; 3) identification of the barriers and facilitators. The analysis started with inductive coding of four interviews (YM). Subsequently, a code tree was developed based on the inductive coding, using the codes from these four interviews, and deductive coding, using the determinants of the MIDI. The combined inductive and deductive coding method was used to ensure no relevant themes were overlooked. Two researchers (EvL and YM) reread the transcripts several times to ensure familiarisation with the data. The preliminary code tree was initially discussed by two researchers (EvL and YM) and subsequently by three researchers (EvL, YM, JvD).

In the second phase, three transcripts were coded by two researchers simultaneously (EvL and YM). The content of the transcripts and associated codes were discussed during the coding process in order to reach an agreement on the coding strategy. Differences in coding were discussed and the code tree was revised if deemed necessary. The remaining transcripts were coded by YM. NVivo Qualitative Research Data Analysis Software, version 12 Pro was used to support the coding process.

Finally, all the codes were analysed to identify the barriers and facilitators within the interviews and categorised per subdomain. The results of the analysis were discussed by three researchers (EvL, YM, JvD). Suggestions for adaptations were made, based on these discussions, in order to specify the MIDI (an elaborate description of the adaptions can be found below). Data saturation within the interviews was reached, meaning that no new barriers, or facilitators or new perspectives emerged from the data in the last interviews.

#### MIDI

Descriptive statistics (mean, standard deviation and percentage) were used for evaluating the barriers and facilitators. A determinant was considered a barrier if ≥ 20% of the healthcare professionals responded with ‘totally disagree’ or ‘disagree’. A determinant was considered a facilitator if ≥ 80% of the healthcare professionals responded with ‘agree’ or ‘totally agree’. This corresponded with the methods of Verberne et al. used when analysing the barriers and facilitators to implementation of paediatric palliative care teams using the MIDI [18]. IBM SPSS Statistics for Windows, Version 26 was used for data analysis. A general inductive approach was used for analysing the comments and answers to the open-ended questions [19].

This study was assessed by the institution’s ethical review board at Gelderse Vallei hospital which judged that formal ethics approval was not required as the Medical Research Involving Human Subjects Act was not applicable. Healthcare professionals participating in the interviews provided written consent.

## Results

### Participants

Fourteen healthcare professionals were purposively invited. Three healthcare professionals declined the invitation due to time constraints (*N* = 1) and absence during the study period (*N* = 2). Subsequently, 11 healthcare professionals participated in the interviews. The mean interview duration was 44 minutes (range 21–60). Thirty-seven of the 48 healthcare professionals responded to the questionnaire (response rate 77.1%). Those participating in the interviews and questionnaire consisted of at least one nurse and one physician from the five outpatient clinics. The characteristics of the participants can be found in Table 1. The majority of the interviewees (9/11) also completed the questionnaire.

**Table 1:** Characteristics of healthcare professionals participating in the interviews and questionnaire based on the MIDI

	Interview (***n*** = 11)	MIDI (***n*** = 37)
**Profession**		
Nurse	6	14
Treating physician^a^	5	23
**Specialty**		
Pulmonology	1	5
Geriatrics	2	9
Cardiology	2	10
Oncology	3	7
Nephrology	3	6
**Experience in current position**		
0–5 years	1	7
6–10 years	4	10
11–15 years	2	12
> 15 years	4	8
**Number of ACP conversations**		
None	0	5
1–5	3	14
6–10	5	13
11–15	1	4
> 15	1	1
Unknown	1	0

^a^ This includes a physician assistant, who functions as a treating physician at the geriatrics department

### Adaptations to the MIDI

In line with other research [18], two open-ended questions were added to the MIDI. These were: “Do you have any tips or suggestions for improving the ACP conversations?”, and; “If you have any comments, please describe them here”. Additionally, respondents were asked to describe other personal benefits and drawbacks in addition to the benefits/drawbacks mentioned in the MIDI. Respondents were also able to add a comment to all questions within the questionnaire. Moreover, several questions were adapted. Within question seven (“relevance for client”), we have differentiated between patients (7a) and their proxies (7b). The answer options to question eight (“personal benefits/drawbacks”) of the MIDI were based on the barriers (disadvantages) and the facilitators (advantages) mentioned during the interviews. Within question nine (“outcome expectations”), we have differentiated between three outcome expectations based on the definition of ACP by Rietjens et al. [1] Within question ten (concerning professional obligation), we have differentiated between the responsibility of the healthcare professional, that of the hospital, and the importance of involving a physician. Question 18 (“To what extent are you informed about the content of the innovation?”) was removed. Within question 20, concerning replacement of staff leaving the organisation in a timely manner, we have differentiated between staff (healthcare professionals) conducting the ACP conversations (20a) and staff supporting in the organisation of the intervention (20b). Within question 23, concerning availability of time, we have differentiated between having sufficient time for introducing and inviting the patient to the intervention (23a), and having sufficient time to conduct the ACP conversation (23b). Within question 25, we have asked about the accessibility of the coordinator instead of asking whether a coordinator was present. Within question 26 (“unsettled organisation”), we added the Covid-19 pandemic (26a) next to the influence of other projects (26b). The revised questionnaire can be found in Table 2.

**Table 2:** Determinants for implementing the MUTUAL intervention as measured by the Measurement Instrument for Determinants of Innovations (*N* = 37)

		N	Mean	SD	Disagree/ totally disagree (%)	Neutral (%)	Agree/totally agree (%)
**Intervention (MUTUAL intervention)**							
1	Procedural clarity: the ACP intervention clearly indicates the activities I should perform and in which order	37	4,05	0,57	0,0	13,5	**86,5**
2	Correctness: the ACP intervention is based on factually correct knowledge	36	3,94	0,53	0,0	16,7	**83,3**
3	Completeness: the ACP intervention provides the information and materials needed to perform the intervention properly	36	4,03	0,70	2,8	13,9	**83,3**
4	Complexity: the ACP intervention is easy for me to use	36	4,00	0,63	2,8	11,1	**86,1**
5	Compatibility: the ACP intervention is a good match for how I am used to working at the outpatient clinic	36	3,86	0,72	5,6	16,7	77,8
6	Observability: the outcomes of using the ACP intervention are clearly observable	36	3,53	0,77	8,3	38,9	52,8
7a	Relevance for patients: the ACP conversations are relevant for my patients	36	4,47	0,56	0,0	2,8	**97,2**
7b	Relevance for their proxies: the ACP conversations are relevant for proxies of the patients	37	4,51	0,61	0,0	5,4	**94,6**
**User**							
8a	Personal benefits: the ACP intervention helps me to improve the quality of care	37	4,11	0,57	2,7	2,7	**94,6**
8b	Personal benefits: the ACP intervention helps me to understand the wishes of my patient	36	4,11	0,62	2,8	5,6	**91,7**
8c	Personal benefits: the ACP intervention improves the connection with my patient	36	3,61	0,73	8,3	27,8	63,9
8d	Personal benefits: the ACP intervention improves follow-up conversations with my patient	36	3,44	0,77	5,6	47,2	47,2
8e	Personal benefits: the ACP intervention saves time in the long term	36	3,56	0,81	8,3	38,9	52,8
8f	Personal benefits: ACP conversations make me feel satisfied	36	3,81	0,71	5,6	19,4	75,0
8g	Personal benefits: the ACP intervention contributes to my personal development	36	3,61	0,84	11,1	27,8	61,1
8h	Personal benefits: the ACP intervention helps me to structure the conversation	36	3,78	0,83	8,3	22,2	69,4
8i	Personal drawback: the ACP intervention is demanding for me	36	3,00	0,76	27,8	44,4	***27,8 (barrier)***
8j	Personal drawback: the ACP intervention raises the workload	36	3,19	0,89	27,8	27,8	***44,4 (barrier)***
*Outcome expectations*							
9a	I expect that the ACP intervention enables the patient to define goals and preferences for future medical treatment and care^a^	33	4,03	0,47	0,0	9,1	**90,9**
9b	I expect that the ACP intervention enables the discussion of goals and preferences with family and healthcare professionals^a^	33	4,21	0,42	0,0	0,0	**100,0**
9c	I expect that the ACP intervention leads to the documentation of treatment preferences^a^	33	4,24	0,50	0,0	3,0	**97,0**
10a	Professional obligation: I feel that the ACP conversations are my responsibility as a professional	33	4,21	0,65	3,0	3,0	**93,9**
10b	Professional obligation: I feel that the ACP conversations are the responsibility of the hospital	33	3,73	0,76	6,1	27,3	66,7
10c	Professional obligation: I feel that the ACP intervention should always involve a physician	33	4,06	0,83	6,1	12,1	**81,8**
11	Patient satisfaction: patients are generally satisfied with the ACP intervention	33	4,09	0,58	0,0	12,1	**87,9**
12	Patient cooperation: patients generally cooperate with the ACP intervention	32	3,66	0,55	3,1	28,1	68,8
13a	Social support: I can count on adequate assistance from my colleagues with the ACP intervention	33	3,91	0,68	0,0	27,3	72,7
13b	Social support: I can count on adequate support from management with the ACP intervention	32	3,28	0,81	9,4	56,3	34,4
14	Descriptive norm: the proportion of colleagues from my specialty who use the ACP intervention^b^	33	4,18	1,31	**39,4**	24,2	36,4
15a	Normative beliefs: physicians from my specialty expect me to use the ACP intervention^a^	33	3,97	0,64	3,0	12,1	**84,8**
15b	Normative beliefs: nurses from my specialty expect me to use the ACP intervention^a^	32	4,00	0,57	0,0	15,6	**84,4**
15c	Normative beliefs: patients expect me to use the ACP intervention^a^	32	3,41	0,61	3,1	56,3	40,6
15d	Normative beliefs: patients’ proxies expect me to use the ACP intervention^a^	32	3,34	0,60	3,1	62,5	34,4
16a	Self-efficacy: Should you wish to do so, do you think you can select and invite patients for the ACP intervention?^a^	33	4,15	0,44	0,0	3,0	**97,0**
16b	Self-efficacy: Should you wish to do so, do you think you are able to use the ACP intervention?^a^	33	4,33	0,54	0,0	3,0	**97,0**
16c	Self-efficacy: Should you wish to do so, do you think you are able to document/register the goals and preferences from the ACP intervention?^a^	33	4,27	0,63	0,0	9,1	**90,9**
17	Knowledge: I know enough to use the ACP intervention correctly	33	3,97	0,47	0,0	12,1	**87,9**
**Organisation**							
19	Formal ratification by management: there are formal arrangements relating to the ACP intervention^c^	37	2,35	0,59	5,4	54,1	40,5
20a	Replacement when staff leave: if employees who are responsible for **conducting** ACP conversations leave the organisation, they are replaced in a timely manner by colleagues who are prepared to have ACP conversations	35	3,14	0,85	**22,9**	37,1	40,0
20b	Replacement when staff leave: if employees that are responsible for **planning** ACP conversations leave the organisation, they are replaced in a timely manner by colleagues who are prepared to plan ACP conversations	34	3,15	0,74	14,7	52,9	32,4
21	Staff capacity: there are enough people in our organisation to use the ACP intervention as intended	34	3,18	0,97	**26,5**	32,4	41,2
22	Financial resources: there are enough financial resources available to use the intervention as intended^c^	35	2,20	0,58	8,6	62,9	28,6
23a	Time available: there is sufficient time available to introduce and invite patients to the ACP intervention at the outpatient clinic	36	3,28	0,97	**25,0**	16,7	58,3
23b	Time available: there is sufficient time available to have the ACP conversations at the outpatient clinic as intended	37	3,41	0,93	18,9	24,3	56,8
24	Material resources and facilities: our organisation provides me with enough materials and other resources or facilities necessary for using the ACP intervention as intended	36	3,69	0,75	11,1	13,9	75,0
25	Coordinator: the coordinator of the ACP intervention is accessible	36	3,94	0,67	0,0	25,0	75,0
26a	Unsettled organisation: the Covid-19 pandemic influences the ACP intervention	37	3,95	0,78	5,4	16,2	***78,4 (barrier)***
26b	Unsettled organisation: there are other projects within the hospital that influence the ACP intervention ^d^	33	1,27	0,45	**27,3 (yes)**		72,7 (no)
27	Information accessible: easy to receive information about the ACP intervention	36	3,75	0,73	8,3	16,7	75,0
28	Performance feedback: within the hospital feedback is regularly provided about progress with the implementation of the ACP intervention	36	3,78	0,76	5,6	25,0	69,4
**Socio-political context**							
29	Regulations: the ACP intervention fits well with existing legislation and regulations	36	3,75	0,50	0,0	27,8	72,2

Numbers in **bold** represent a barrier reported by a HCP (≥ 20% disagrees/totally disagrees) or facilitator reported by a HCP (≥ 80% agrees/totally agrees). 8i, 8j, and 26a are barriers since the determinant concerns a negative response. The number of participants (N) answering the questions of the MIDI differs due to incomplete responses

*ACP* advance care planning, *MUTUAL ***MU**ltidisciplinary **T**imely **U**ndertaken **A**dvance Care P**l**anning, *HCP* Healthcare professional

^a^ Answer categories were divided into 1 ‘most definitely not’, 2 ‘definitely not’, 3 ‘perhaps not, perhaps’, 4 ‘definitely’, 5 ‘most definitely’

^b^ Answer categories were divided into 1 ‘not a single colleague’, 2 ‘almost no colleague’, 3 ‘a minority’, 4 ‘half of colleagues’, 5 ‘a majority’, 6 ‘almost all colleagues’, 7 ‘all colleagues’

^c^ Answer categories were divided into 1 ‘no’, 2 ‘I don’t know’, 3 ‘yes’

^d^ Answer categories were divided into 1’no’, 2 ‘yes’

### Barriers and facilitators

An overview of the barriers and facilitators identified by the modified MIDI can be found in Table 2. Eight barriers and 20 facilitators were identified. The barriers to implementation of the MUTUAL intervention were identified within the user (three barriers), and the organisation domain (five barriers). The facilitators were identified within the intervention (six facilitators) and the user (14 facilitators) domain. The content of the interviews and the answers to the questionnaires are used for clarification and illustration of the barriers and facilitators throughout the result section. Illustrating quotes from the interviews are presented in Table 3. The barriers identified, and the associated suggestions for improvement, can be found in Table 4. Since the facilitators are identified within the intervention and user domain (the first two domains within the MIDI) we will present the facilitators first.

**Table 3:** Illustrating quotes from interviews

*Quote (number)*	*Related MIDI determinant*	*Quote*
*#1*	*7a*	*“I believe the conversations are very valuable since they help you to get to know the patient and what matters to him/her. Patients really appreciate it [having the ACP conversations]. Personally, I also like this and the fact that they say they appreciated talking about this [what is important to them] […].” (int 1., nurse)*
*#2*	*7a*	*“The additional value of the ACP intervention is that it creates a starting point for discussion for later conversations. […] and it can be useful to refer to in later conversations: remember, we have talked about this and this is what you mentioned […]. The multidisciplinary setting helps [getting the wishes of the patient known] since the nurse is able to retrieve other information using the preparatory questionnaire than I am able to retrieve during a normal outpatient encounter with the patient […].” (int.4, physician)*
*#3*	*7a*	*“Some people are not realistic, and their wishes contain many contradictions. [For example] If a patient states that it is essential to have a certain quality of life, and at the same time still wishes to be admitted to an intensive care unit [if necessary]. If you think [as a healthcare professional] that these wishes are conflicting, then it is your duty to inform the patient about what admission to an intensive care unit looks like, what the potential consequences are, and what the chances of recovery are.” (int 2., nurse)*
*#4*	*8a*	*“Sometimes when we are asked to think [in our consultative function] along with patients on the ward, for example whether a patient has a delirium, [if] it turns out that the patient does not want to be treated anymore […] then we advise an ACP conversation or ask the palliative care team for advice.” (int.9, nurse)*
*#5*	*8f*	*“It is fulfilling to have these kinds of [ACP] conversations, because you are able to help a patient in the process [of ACP], in becoming more aware […], because [you are also able to help] if questions arise concerning end of life matters.” (int.7, nurse)*
*#6*	*8 g*	*“It is something you have to grow into, to have these kinds of conversations, to ask the right open questions, to notice the right things. A lot of it is subjective, it’s about what you see and what you feel. That’s something you must get acquainted with.” (int.2, nurse)*
*#7*	*9a* *9b* *9c*	*“Even if patients did not fill in the preparatory questionnaire, they have read the questions and thought about it. […] It [the preparatory questionnaire] helps the patient to understand the goal of the conversation.” (int.1, nurse)*
*#8*	*23a*	*“I only see patients once or twice a year [at the outpatient clinic]. Those people have a lot of questions and there are so many things they want to know. To say, in addition, in that situation, let’s talk about ACP … you just don’t get around doing that. There is simply no time for it.” (int. 11, physician).*
*#9*	*20a* *23b*	*“There was a time when one nurse had just retired and the other one was almost completely on her own. That might have unconsciously made you feel like “okay, we’re not going to have a formal ACP conversation, I will just do it [discuss treatment preferences] myself.” (int. 5, physician)*
*#10*	*6*	*“The [ACP] letter ‘disappears’ in the medical healthcare system, it just falls away. I am not sure if everyone [the involved healthcare professionals] is informed of the existence of the ACP letter. If, for example, a patient is admitted for surgery nine months after the ACP conversation, then I am not sure if the letter will be noticed and read.” (int.7, nurse)*
*#11*	*6*	*“But at some point, the patient goes back to the general practitioner. We never receive information afterwards. I have only had that [received information] once, that was precious. [I heard that] the ACP letter had been helpful and that the patient didn’t get resuscitated and he died the way he wanted to. I only got that feedback once. I think it would be nice for me and my colleagues to know that what we are doing [having ACP conversations] is helpful in the domestic atmosphere. It helped me to hear that from the general practitioner.” (int.1, nurse).*
*#12*	*23b*	*“What is important [for organising the ACP conversations], is the support from nurses, they have to be able to spend a certain amount of time per week having these conversations […]. The organisation should create the conditions, [to enable implementation of the ACP intervention]: the time, and support from nurses.” (int. 10, physician).*

**Table 4:** Barriers and suggestions for improvement based on interviews and comments/answers to open-ended questions contained within the MIDI

Barrier	Domain and determinant of MIDI	Suggestions for improvement
1. Intervention is perceived as demanding	User – 8i	1) Create a memory card with supporting questions for the different theme’s discussed within the ACP conversation. 2) Have manageable expectations within the ACP conversation: “*You can’t always reach your goal, but that doesn’t mean you did not have a good conversation. Most important is that the patient gets insight into what matters most and you start the process of ACP.”* 3) The development of skills is important and demands the ability for self-reflection. It helps to include other healthcare professionals in this process.
2. Intervention raises workload	User – 8j	1) Optimise administrative support (e.g., for planning ACP conversations, and support for documentation). 2) Create a backup system for having ACP conversations (e.g., nurses from the palliative care team could have ACP conversations at other outpatient clinics in case of a lack of capacity).
3. Less than half to not a single colleague from my specialty use/uses the ACP intervention	User – 14	1) Frequent (e.g., monthly) reminders to improve awareness for healthcare professionals. 2) Raise awareness within patients and their proxies. 3) Embed, structurally, ACP conversations within usual care, and create a routine.
4. No replacement of staff in a timely manner	Organisation – 20a	1) Prioritise replacing healthcare professionals who are able to have ACP conversations and support the timely preparation of healthcare professionals to enable them to have ACP conversations.
5. Insufficient capacity	Organisation - 21	2) Offering patients ACP conversations should be positioned as a standard medical procedure.
6. Insufficient time for introducing and inviting patients for an ACP conversation	Organisation – 23a	1) Explicate additional value and importance of having ACP conversations. 2) Prioritise the introduction of ACP conversations to patients during regular outpatient clinic visits. 3) Expand the means by which patients can be invited for an ACP conversation. This can include having the opportunity for other healthcare professionals (e.g., nurses or paramedical staff on wards or at outpatient clinics) to be involved, structurally, in the selection process. Also incorporate, structurally, discussions surrounding the selection of patients at other meetings, including multidisciplinary ones.
7. Influence of Covid-19	Organisation – 26a	1) No specific suggestions for improvement.
8. Other projects	Organisation – 26b	1) No specific suggestions for improvement.
Other tips/suggestions	Not applicable	1) Encourage general practitioners to have ACP conversations and share advance directives with treating physicians and incorporate these [advance directives] structurally into referral letters. 2) More support from management. 3) More awareness for palliative care in general.

#### Facilitators

The facilitators related to the ACP intervention were its “clarity”, “correctness”, “completeness” and “simplicity”. Additionally, facilitators concerning the intervention were “relevance for patients” (97.2% agreed) and “relevance for their proxies” (94.6% agreed). Interviewees expressed the relevance of the ACP conversations by stating that these were believed to be valuable and that patients appreciated having them (Table 3, quote 1). Moreover, ACP conversations are mentioned as a means of helping to get to know patients better and understanding what is most important to them (quote 1 and quote 2). These conversations were also believed to help in making informed, and shared decisions (quote 3).

Fourteen facilitators were identified in the user domain. These included two personal benefits: the ACP intervention helps healthcare professionals to improve the quality of care (94.6% agreed) and it helps to understand patient wishes (91.7% agreed). Within the interviews, ACP conversations are reported to affect patient encounters beyond the MUTUAL intervention (quote 4), hereby improving the quality of care.

Within the MIDI, the advantages 8f (“make me feel satisfied”) and 8 g (“contribute to personal development”) have not been identified as facilitators (75.0 and 61.6% agreed, respectively). In the questionnaire, the advantage of personal development is described as follows: *“ACP conversations contribute to my personal development, conversing about the value of illness and health is meaningful in all encounters with patients.”* This corresponds with the interviews, in which ACP conversations are mentioned as being fulfilling (quote 5) and contributing to personal development (quote 6). A separate analysis of responses from nurses in the MIDI showed that 85.7% agreed that the ACP conversations are satisfying and 85.7% also agreed that these contribute to their personal development. Another advantage mentioned within the questionnaire is that the ACP intervention “*creates a fixed moment where patient and proxies are able to discuss important issues*”. Other personal benefits of the ACP intervention mentioned during the interviews include that they “*improve patient connection*”, “*improve follow-up conversations*”, “*save time in the long run*”, “*make me feel satisfied*”, and “*help in structuring ACP conversations*”.

Three facilitators in the user domain were related to outcome expectations. Healthcare professionals expect that the ACP intervention “enables the patient to formulate goals and preferences for future medical treatment and care” (90.9% agreed), “enables the discussion of goals and preferences with family and healthcare professionals” (100.0% agreed) and “leads to the documentation of treatment preferences” (97.0% agreed). Nine other facilitators fell within the determinants “professional obligation”, “patient satisfaction”, “normative beliefs”, “self-efficacy”, and “knowledge”. Of the respondents, 93.9% agreed that “ACP conversations are the responsibility of their profession” and 81.8% agreed that “a physician should always be involved in the ACP intervention”. Among respondents, 87.9% agreed that “patients are generally satisfied with the ACP intervention” and 87.9% of the healthcare professionals agreed that they “have sufficient knowledge to use the ACP intervention correctly”. Interviewees mentioned the relevance of various elements of the MUTUAL intervention. The preparatory questionnaire, for instance, was mentioned as a facilitator for the ACP conversations since it helped the patient to understand the goal of the conversation and hereby facilitated the conversation (quote 7).

#### Barriers

Three barriers lay within the user domain. Firstly, the intervention is perceived as demanding by 27.8% of the healthcare professionals and raises the workload according to 44.4%. Additionally, 39.4% of the participants reported that “less than half to not a single colleague from my specialty use/uses the ACP intervention”. The quotes from the MIDI illustrate the potential burden on healthcare professionals, and reflect on the alternatives. One healthcare professional commented: *“Documentation of the ACP conversation takes a lot of time. There is no time reserved within the outpatient clinic schedule for documentation. It [the ACP intervention] takes a lot of time, including preparation, documentation, etc.”,* and: “*Time investment [is high], however, otherwise these [ACP] conversations should take place without reserved time, which is less desirable.”* Interviewees mentioned that gaining more experience makes the ACP conversations less demanding. Uncertainty concerning who should be responsible for initiating ACP conversations, and the limited cooperation between the general practitioner and hospital physicians, were mentioned in the interviews as potential barriers. Moreover, it was mentioned that the division of tasks seems less clear when patients have multiple comorbidities requiring them to be seen by several physicians.

The most important barrier mentioned during the interviews was a lack of time. It was suggested that this impedes the implementation of the intervention in several ways. For example, healthcare professionals mentioned that there was not sufficient time to introduce and invite the patient to an ACP conversation during the regular visits at the outpatient clinic (quote 8). This is confirmed by the results of the MIDI which showed that 25.0% of the healthcare professionals disagreed that there is sufficient time for introducing and inviting patients to such ACP conversations at the outpatient clinic. When analysing the results separately for the physicians who are responsible for introducing and inviting patients, this barrier is even more outspoken (39.1% disagreed). Moreover, 22.9% of the healthcare professionals disagreed that there is replacement in a timely manner when healthcare professionals responsible for conducting ACP conversations leave (quote 9). Furthermore, 26.5% disagreed that there is sufficient staff capacity for implementation of the ACP intervention. Additionally, planning the ACP conversations can cause problems. This is reflected in a comment in the questionnaire that read, *“Planning a conversation in a multidisciplinary setting (with nurse and physician) is sometimes difficult”.* Moreover, it is mentioned that it is more difficult to plan ACP conversations if no regular timeslots have been reserved within the normal schedule of the outpatient clinic.

Two barriers are related to there being an “unsettled organisation”. Among the participants, 78.4% of the participants agreed that Covid-19 influences the ACP intervention. For example, healthcare professionals mentioned that they were reluctant to invite patients to have ACP conversations due to the risk of exposure to Covid-19. Additionally, 27.3% of the participants responded that other projects within the hospital influence the ACP intervention.

Another barrier mentioned in the interviews is the uncertainty of the effect of the ACP intervention (quote 10). Healthcare professionals explain that they are not aware of the continuation of the patient journey and neither are they convinced that other healthcare professionals are sufficiently aware of the documentation of the ACP conversations. However, the lack of effect of ACP is not a barrier within the MIDI. Of the healthcare professionals, 8.3% disagreed that the outcome of using the ACP intervention is clearly observable (52.8% agreed). However, the interviewees mentioned that if they are informed of the positive effect of the ACP intervention, then this is a facilitator and motivator (quote 11). All healthcare professionals report in the interviews that a lack of awareness and a gradual drop in attention afforded to the ACP intervention once it has begun, is a barrier to the implementation of the ACP intervention. Moreover, within the interviews and questionnaires, the importance of organisational support is stressed (quote 12).

Several suggestions for improving the implementation of the MUTUAL intervention were made. These included making ACP conversations less demanding (e.g., help with skills development) and decreasing the workload (e.g., administrative support). A lack of time was mentioned as a barrier in several ways, including the lack of time to introduce and invite patients. Various suggestions to expand the way patients are to be invited for an ACP conversation were made. These included creating an opportunity for other healthcare professionals, for example nurses or paramedical staff on wards or at outpatient clinics to be involved, structurally, in the selection process. Another suggestion was to incorporate discussions on the selection of patients into the structure of other meetings, including multidisciplinary ones. The barriers identified by the MIDI and the associated suggestions for improvement, can be found in Table 4.

## Discussion

This study aimed to identify the barriers to, and facilitators for, the implementation of the recently developed MUTUAL intervention at the outpatient clinic. Overall, our results show that healthcare professionals positively evaluate the MUTUAL intervention and are very receptive to implementing the MUTUAL intervention. However, taking into account the suggestions for improvement may enhance further implementation. All the facilitators were identified in the intervention (six facilitators) and user (14 facilitators) domains. Healthcare professionals agreed that the elements of the MUTUAL intervention are clear, correct, complete, and simple, and they are convinced that it is relevant for patients and their proxies. Moreover, several personal benefits are revealed and the intervention is expected to achieve the goals of ACP. The main barriers to the successful implementation of the MUTUAL intervention are identified within the user domain (three barriers) and within the organisation domain (five barriers). There are no barriers related to the intervention itself. Yet, the intervention is perceived as demanding by approximately a quarter of the participants and raises the workload according to almost half of them. Barriers related to the organisation include the inadequate replacement of staff, insufficient staff and insufficient time to introduce and invite patients to the ACP intervention. Additionally, Covid-19 and other projects within the hospital, negatively influence the implementation of the MUTUAL intervention. Several suggestions for the improvement of the implementation of the MUTUAL intervention were made.

The barriers found within our study are related to the organisational domain of the intervention and not to the intervention itself. Hafid et al. investigated the implementation of ACP conversations in primary care based on the structured Serious Illness Conversation Guide [20]. The study by Hafid et al. also identified logistical challenges for the implementation of ACP that corresponded to our results. However, the study by Hafid et al. also identified barriers that were related to their intervention, whereas our study did not find any barriers related to this ACP intervention.

In the study by Hafid et al. the identification of appropriate patients for ACP is mentioned as a barrier [20]. The identification of patients was not seen as a barrier in our study. Using the surprise question (SQ) “Would I be surprised if this patient were to die in the next 12 months?” seemed helpful for the identification of patients since it functioned as a reminder for healthcare professionals to invite patients for the ACP intervention, especially for healthcare professionals not experienced with ACP conversations. A recent systematic review and meta-analysis showed that the surprise question has an estimated sensitivity of 71.4% and a specificity of 74.0% in predicting death, and may be a useful tool for initiating ACP [21]. Our study showed that barriers to the selection process shifted from the identification of patients to the invitation of patients since lack of time hindered inviting patients to the MUTUAL intervention. Physicians did not have sufficient time to introduce and invite patients during regular visits to the outpatient clinic.

Our study showed that a lack of time influences several elements of the MUTUAL intervention, in addition to those related to introducing and inviting patients to the ACP intervention. Moreover, it was mentioned that planning the ACP conversation can be difficult due to a lack of time in general, or due to issues concerning the need for simultaneous planning of the nurse and physician for an ACP conversation. Knowing the various ways in which a lack of time can impede the implementation of ACP might help overcome these barriers in the future.

A frequently mentioned barrier to engagement in ACP is the lack of preparation of patients and their proxies [20, 22]. The preparation of patients is part of the MUTUAL intervention and consists of an information folder and a preparatory questionnaire. In our study, the preparation of the patient was observed to contribute to the patient’s awareness of the goal of the conversation. Furthermore, patients were encouraged to think about preferences in advance, facilitating the ACP conversation. Our study showed that this preparation, a part of the MUTUAL intervention, facilitates the process of ACP.

The study by Hafid et al. identified the discussion of their prognosis with patients as a barrier for allied health professionals such as nurse practitioners, registered nurses, and social workers, in facilitating ACP conversations [20]. By introducing the multidisciplinary approach, that is by the treating physician joining the ACP conversation, we hoped, and we think on reasonable grounds, that this will overcome this barrier for nurses. Whether we succeeded in this, requires further research. The scripted conversation in a multidisciplinary setting was described as valuable, and the interaction between nurses and physicians was described as pleasant and effective. Almost all healthcare professionals agreed that ACP conversations belong to their profession and the majority agreed that a physician should be involved in ACP conversations. Moreover, insufficient knowledge about the patient’s prognosis was not identified as a barrier in this study. Our study showed that the multidisciplinary approach of the MUTUAL intervention is perceived as valuable and feasible despite the intensified role of the physician compared to earlier ACP interventions.

Within this study, secondary care health professionals agreed that they play an important role in ACP, and that ACP conversations should also take place in the hospital. At the same time, they underpinned the importance of further incorporation of ACP into primary care and the importance of collaboration with primary care physicians. However, uncertainty concerning who should be responsible for initiating ACP conversations, and the limited cooperation between general practitioners and hospital physicians were mentioned as barriers during the interviews. These findings emphasise the importance of incorporating ACP into primary care and the need for increased collaboration in the structure of healthcare throughout its supply chain. A barrier mentioned in the interviews is the uncertainty of the effect of ACP. Interviewees link the effect to the documentation of the intervention. The documentation of the ACP process is recognised as an important step in the MUTUAL intervention. This element of the MUTUAL intervention was considered to be a complete and a clear representation of patient preferences. Based on the MIDI, we cannot conclude that a lack of clarity as to the effect of the intervention is a barrier or facilitator. However, the interviews, comments, and open-ended questions suggest that informing healthcare professionals of the effect of the ACP intervention might encourage implementation. Hence, collaboration between the primary and secondary healthcare might not only be beneficial for patients, by achieving more goal-concordant care, but might also be a facilitator in the implementation of ACP interventions. Therefore, this should receive the necessary attention.

### Implications for practice

ACP is seen as a promising solution for non-beneficial care [23], which is also associated with burn-out among healthcare workers [24, 25]. Situations involving futile and inadequate care at the end of life have been shown to contribute more to nurses’ moral distress than any other aspect of care and are linked to burn-out and staff leaving their jobs [26]. Hence, ACP could contribute to lower moral distress in healthcare professionals. Piers et al. suggest that the active involvement of nurses in end of life decision making could lead to less moral distress in nurses and at the same time benefit patient care [26]. Our study revealed that nurses experience their role within the MUTUAL intervention as fulfilling. The multidisciplinary setting, and the role of the nurse, have been shown to be of utmost importance. As stated before, getting positive feedback on the effect of the ACP conversations potentially increases this fulfillment. Hence, engaging nurses in ACP conversations might positively influence the way nurses experience their work and has the potential to reduce burn out and the experience of moral distress. However, this was outside the scope of this study and requires further research.

As stated above, barriers found in this study are mainly related to the organisational domain. In general, financial resources and formal ratification are relevant for implementing interventions. However, in our study these factors have not been directly identified as barriers or facilitators by using the MIDI, nor have they been explicitly mentioned as barriers during the interviews. However, barriers and facilitators might change over time and limited financial resources, and/or time constraints, might become barriers when there is an increase in ACP conversations in an organisation. The importance of sufficient resources is also reflected in the results of Hafid et al.: 50% of the healthcare professionals stated that available resources in primary care are not adequate for implementation [20]. Within the interviews and the MIDI, it is suggested that the policy of the organisation should reflect the importance of the implementation of ACP. Piers et al. agree in their recommendations for implementation of ACP in dementia care. They state “*integrate ACP into the mission and policy of the organisation and embed in the organisation culture.*” [27] The aforementioned lack of time is evidently related to the availability of financial resources, including financial ones. Hence, embedding ACP in the mission, vision, and policy of organisations, should receive the necessary attention for ACP interventions to be successfully implemented.

## Strengths and limitations

This is a mixed-methods study using both interviews and a validated method for evaluating the implementation of interventions - the MIDI. We tailored the MIDI based on the qualitative data from the interviews. The content of the interviews and the questionnaire, both the comments and the answers to the open-ended questions, are used for clarification and illustration of the barriers and facilitators. Using both data from interviews and a questionnaire expands understanding, while at the same time being comprehensive. Since the MUTUAL intervention was implemented at various outpatient clinics, we were able to study barriers and facilitators with patients with various underlying diseases. This is in contrast to most other studies that only focus on one type of disease. Several limitations to this study need mentioning. The number of healthcare professionals participating in the interviews as well as in the questionnaire is limited. However, due to the relatively high response rate (77.1%) to the questionnaire, we think this study gives a fair representation of the main barriers and facilitators in this setting. Additionally, this study is limited to five outpatient clinics within one hospital. Hence, the results of the study cannot be generally applied to other populations or care settings. Healthcare professionals who have yet to participate in the MUTUAL intervention have also been included. This suggests that it was not only healthcare professionals supportive of ACP who participated in this study, thus expanding the degree to which it can be generally applied. Furthermore, it should be mentioned that determinants of the MIDI that did not meet the requirements for a barrier or facilitator according to the criteria, could potentially still have an influence on implementing the ACP intervention. By integrating the qualitative data with the quantitative data, we have sought to address the most important barriers and facilitators influencing implementation.

## Conclusion

This study shows that healthcare professionals positively evaluate, and are very receptive to implementing, the MUTUAL intervention. The main barriers involve the organisational domain, including the inadequate replacement of staff, insufficient staff, and insufficient time to introduce and invite patients to an ACP conversation. Furthermore, attention needs to be given to providing adequate support for the healthcare professional in order to decrease the workload of the ACP intervention. Our results stress the importance of organisational support for successful implementation of the MUTUAL intervention. It is also important that ACP is embedded in the structure of healthcare throughout its supply chain. Taking into account the suggestions for improvement may enhance further implementation of ACP, hereby potentially improving person-centered care.

## Supplementary Information

**Additional file 1.** Interview guide for nurses.

**Additional file 2.** Interview guide for physicians.

## Data Availability

The datasets analysed during the current study are not publicly available due to participant privacy reasons but are available from the corresponding author on reasonable request.

## Abbreviations

ACP: Advance Care Planning
HCP: Healthcare professional
MIDI: Measurement Instrument for Determinants of Innovations
MUTUAL: MUltidisciplinary Timely Undertaken Advance Care PLanning
SQ: Surprise Question (“*Would I be surprised if this patient were to die in the next 12 months*?”)
