# Supplementary material for: Barriers and facilitators for healthcare professionals to the implementation of Multidisciplinary Timely Undertaken Advance Care Planning conversations at the outpatient clinic (the MUTUAL intervention): a sequential exploratory mixed-methods study
Source: BMC Palliat Care. 2023 Mar 15;22:24. doi: 10.1186/s12904-023-01139-y (PMC10015131; doi:10.1186/s12904-023-01139-y)
Supplement: Supplementary file 1 — Additional file 1. Interview guide for nurses. [file 12904_2023_1139_MOESM1_ESM.docx]

# Supplementary File 1 – Interview guide for nurses

General

In 2018, a pilot study consisting of ACP conversations at the outpatient clinic was conducted at Gelderse Vallei Hospital. Subsequently, in 2019 these conversations were implemented, structurally, at five outpatient clinics.

- How many ACP conversations have you conducted since the start of this project?
- What is the added value of the ACP conversations at the outpatient clinic for patients and healthcare professionals?

The purpose of the ACP conversations at the outpatient clinic is to enable the patient to formulate goals and preferences for future medical treatments and care, to discuss these goals and preferences with family and healthcare professionals, and to make concrete treatment agreements if possible. These agreements can always be changed.

- Are you able to achieve these goals?
  - Why, or why not?

Patient selection

- Usually, the cardiologist/nephrologist/pulmonologist/

geriatrician/oncologist selects patients who are eligible for an ACP conversation using the surprise question (SQ, “would you be surprised if this patient died within a year”). Are there other ways patients are invited for an ACP conversation?

- - If yes, how?
  - Do you use the SQ?
- In your opinion, are there patients who are invited, or are not invited, incorrectly for an ACP conversation?
  - If yes, what is this caused by?
- How could the selection of patients be promoted?

Preparation

- How do you prepare for an ACP conversation?
- How did you experience the training?
- Patients receive a preparatory questionnaire prior to the conversation:
  - To what extent is this questionnaire completed by the patients?
  - Do you think the preparatory questionnaire adds value?
    - Why, or why not?

ACP conversation

- How do you experience conducting ACP conversations at the outpatient clinic?
- What helps you in conducting ACP conversations?
  - What factors contribute to a positive outcome of the conversation?
- What barriers do you experience when conducting ACP conversations?
  - What patient-related factors obstruct conducting ACP conversations?
    - *Uncertainties and complexity of the course of the disease*
    - *Resistance from the patient or their proxy*
    - *Unclear wishes of the patient*
    - *Limited connection with the patient*
    - *Limited knowledge of the patient concerning course of the disease*
    - *Culture or religion*
  - What personal factors obstruct conducting ACP conversations?
    - *Uncertainty towards their own skills*
    - *Sensitivity towards the topic and their own experiences*
- In what way could the barriers previously mentioned be fully or partially solved?
- To what extent do you use the conversation manual?
  - Does the manual help?
  - If yes: how?
  - If no: how should the manual be improved?

Documentation

- How do you experience the process of documentation?
- What factors help with the documentation?
- Do you experience problems?
  - If yes, what kind of problems?
- Do you feel that patient’s wishes are properly documented?
  - Why, or why not?
- To what extent do you experience that treatment preferences, discussed during the conversation, are followed up by other healthcare professionals?

Evaluation

- How do you experience the evaluation following the conversation?
- How satisfied are you, on average, after a conversation?
- Do you feel that patients and their proxies are satisfied with the conversation, both directly after the conversation and in the long run?

Organisation

- How do you experience the organisation of the ACP conversations at the outpatient clinic?
- To what extent is the procedure for planning and conducting the ACP conversation clear?
- Is information/material missing?
  - If yes: what is missing?
- What positive factors regarding the organisation of the ACP conversations can you mention?
  - *Saving time in the long run*
- Are there limiting factors concerning the organisation?
  - *Mutual division of tasks*
  - *Time*
  - *Funding*
  - *Collaboration with general practitioners*
- How could the organisation of the ACP conversations be improved?
